# Statement of Retraction: STAT3-induced upregulation of long noncoding RNA HNF1A-AS1 promotes the progression of oral squamous cell carcinoma via activating Notch signaling pathway

**DOI:** 10.1080/15384047.2026.2740303

**Published:** 2026-09-29

We, the Editors and Publisher of *Cancer Biology & Therapy*, have retracted the following article:

Liu, Z., Li, H., Fan, S., Lin, H., & Lian, W. (2019). STAT3-induced upregulation of long noncoding RNA HNF1A-AS1 promotes the progression of oral squamous cell carcinoma via activating Notch signaling pathway. *Cancer Biology & Therapy*, *20* (4), 444–453. https://doi.org/10.1080/15384047.2018.1529119

Following publication, concerns were identified regarding the image integrity of Western Blot Figures 2D, 3B, and Immunofluorescence Figure 3 C.

Further investigation by the Publisher identified that this study used the contaminated Tca8113 cell line. Following review by the publisher and the journal Editor, it was decided that the results of the study can no longer be relied upon. When approached for an explanation, the authors did not respond to our queries.

As verifying the validity of published work is core to the integrity of the scholarly record, we are therefore retracting the article. The corresponding author has been informed.

We have been informed in our decision-making by our editorial policies and the COPE guidelines.

The retracted article will remain online to maintain the scholarly record, but it will be digitally watermarked on each page as ‘Retracted’.

